# Hypotheses on Mechanisms Linking Cardiovascular and Psychiatric/Neurological Disorders

**DOI:** 10.1155/2009/197132

**Published:** 2009-10-26

**Authors:** Hari Manev

**Affiliations:** The Psychiatric Institute, Department of Psychiatry, University of Illinois at Chicago, Chicago, IL 60612, USA

This special issue of *Cardiovascular Psychiatry and Neurology *marks its first year since we launched the open access journal dedicated to publishing original preclinical/basic and research on biological mechanisms of and treatments for co-occurring cardiovascular disorders and disorders of the central nervous system (CNS). As suggested earlier (by the paper entitled “The heart-brain connection begets cardiovascular psychiatry and neurology” by Hari Manev), at least three scenarios could be at play in these disorders: (1) the primary pathological mechanism in the cardiovascular system triggers a nervous system dysfunction, (2) the primary pathological mechanism in the nervous system triggers a cardiovascular pathology by disrupting the physiological links between the two systems, and (3) the primary pathology is in a biological mechanism that is normally operative in both the nervous and the cardiovascular systems, thus causing the cooccurrence of pathologies, that is, the cooccurring pathologies share a pathobiological mechanism but do not necessarily cause each other. The ongoing research explores the validity of these conceptual scenarios.

Whereas the evidence for the association of cardiovascular and CNS disorders derives primarily from epidemiological studies, direct evidence and experimental data are just beginning to emerge to support this notion. Difficulties in directly demonstrating and explaining the cooccurrence of cardiovascular and CNS disorders lie in part in the limitations of their respective animal models (in particular animal models of psychiatric disorders) and in part in conceptual uncertainties regarding the mechanisms linking heart-brain pathologies that hamper respective clinical research. Historically, bold hypotheses along with serendipitous observations have been instrumental in breakthrough discoveries.

This collection of hypotheses on mechanisms linking cardiovascular and psychiatric/neurological disorders has been assembled with the goal of stimulating discussions in this field of bioscience and medicine. The postulated mechanisms range from molecular targets and pathways (the papers by Sébastien S. Hébert entitled “Putative role of microRNA regulated pathways in comorbid neurological and cardiovascular disorders”, by David P. Gavin and Rajiv P. Sharma entitled “Chromatin from peripheral blood mononuclear cells as biomarkers for epigenetic abnormalities in schizophrenia”, by Neil R. Smalheiser entitled “Do neural cells communicate with endothelial cells via secretory exosomes and microvesicles?” by Stephen D. Skaper and Pietro Giusti entitled “P2X7 receptors as a transducer in the cooccurrence of neurological/psychiatric and cardiovascular disorders: A hypothesis”, by Charles D Nichols entitled “Serotonin 5-HT2A receptor function as a contributing factor to both neuropsychiatric and cardiovascular diseases” by Hu Chen entitled “Possible role of platelet GluR1 receptors in comorbid depression and cardiovascular disease”, by Susana Roque, Margarida Correia Neves, Ana Raquel Mesquita, Joana Almeida Palha, and Nuno Sousa entitled “Interleukin-10: A key cytokine in depression?” by Jin Chu and Domenico Praticò entitled “The 5-Lipoxygenase as a common pathway for pathological brain and vascular aging”, by Jane Pei-Chen Chang, Yi-Ting Chen, and Kuan-Pin Su entitled “Omega-3 polyunsaturated fatty acids (n-3 PUFAs) in cardiovascular disease (CVD) and depression: The Missing Link?” by Janusz K. Rybakowski entitled “Matrix metalloproteinase-9 (MMP9)—a mediating enzyme in cardiovascular disease, cancer, and neuropsychiatric disorders”, and by Thomas Müller entitled “Possible treatment concepts for the Levodopa related hyperhomocysteinemia”) to system functioning (the papers by Britta A. Larsen and Nicholas J. S. Christenfeld entitled “Cardiovascular disease and psychiatric comorbidity: The potential role of perseverative cognition” and by Hadar Shalev, Yonatan Serlin, and Alon Friedman entitled “Breaching the blood-brain barrier as a gate to psychiatric disorder”). We hope that exploring the hypotheses presented in this special issue and, more generally, focusing on the putative mechanisms of the cooccurrence of cardiovascular and CNS disorders will help us to identify unexpected and novel therapeutic targets and to ultimately lead to better treatment of both types of disorders.

*Hari Manev*

